# The Medical Work of the Local Government Board

**Published:** 1893-10-21

**Authors:** 


### The Medical Work of the Local Government Board.

### VACCINATION.

The report of the Medical Officer of the Local Government Board is very strictly kept to a bare record of facts. Inferences and opinions are conspicuous by their absence, and yet this seemingly bald digest of results is full of material for thought. It is much to be regretted that the popular opinion that "blue books" are dull (though this report is bound in yellow) prevents such volumes as this being read by the public, whom, if they only thought of it, it very closely concerns. For our own part, we think it will not be wholly useless to tell that portion of the public which reads our pages something of what the Government of their country does to preserve public health, as evidenced by the report of the Local Government Board.

The one matter which it is generally known that the State takes in hand is vaccination. As a nation we believe in vaccination. There are, it is true, exceptional individuals who do not. Among these may be included nearly all mothers of children under six months old, who don't see why their babies should be made feverish and cross for the sake of escaping the risk of a disease which, as its ravages become fewer every year, seems to them a very remote danger. We are certainly becoming more sceptical than our parents were as to the value of vaccination. The reason is not far to seek. We see comparatively little small-pox. In the report of a medical missionary in Africa, the writer favourably compares the readiness with which native mothers brought their children to be vaccinated, with the indifference and even antagonism to vaccination which prevails in some localities in Britain. But if English mothers saw their children swept away by small-pox in the numbers which that disease still slays among the unvaccinated of Central Africa, they would gladly try anything that promised to save them from the attack of the destroyer.

This scepticism with regard to the value of vaccination is certainly growing among us. The birth and vaccination returns given in this report, published in 1893, are for no nearer date than 1889; for such numbers cannot be calculated in a day; but we doubt if later statistics would show any change in the national tendency. In 1889, then, there were 885,909 children born in England and Wales, of whom 707,161 were registered, at the time the return was made, as successfully vaccinated. This leaves 178,748, or 20 per cent. of the total births, to be accounted for. Of these 88,995 are registered as having died unvaccinated, 1,758 as being insusceptible of vaccination, 2 as having had small-pox, and thereby acquiring the right to exemption from the minor disease; and 13,366 as having their vaccination postponed by medical certificate. This leaves 74,627 children unaccounted for. Most of them are recorded as "removed" or "not to be traced," and though it is possible enough that some of these may have been vaccinated elsewhere than in the district they were born in, it is not likely that they formed any large proportion of the whole. We shall not greatly err, therefore, in reckoning all these children as unvaccinated. They make thus 9·4 per cent. of the whole number of children born. There is something not altogether satisfactory, to those who believe in vaccination, in the thought of 9 per cent. of the children of the nation not having undergone the preventive inoculation; but it is still less satisfactory to note that the dislike to vaccination, as thus evidenced, has increased since the passing of the Vaccination Act of 1871. In 1872 8·8 per cent. of the children born in the metropolis remained unvaccinated, and 4·5 per cent. of those born in the rest of England. In 1889 the proportions were 11·6 per cent. of metropolitan and 9·6 per cent. of provincial children. The metropolis has shown itself somewhat capricious as to vaccination, first being increasingly favourable to vaccination up to 1881, when the proportion of the unvaccinated was only 5·7 per cent., and since then having a steadily increasing number who manage to escape the so-called compulsory operation. In the rest of England, on the other hand, the distaste has grown more slowly, but more steadily. From 4·5 per cent. in 1872 the proportion of the unvaccinated fell in the provinces to 3·8 in 1875. In 1876 it rose to 4 per cent., and from that date it has slowly increased. It was not till 1884 that it reached 5.3 per cent., but since then the development of the rate of evasion has been more rapid, the successive percentages being, for 1885, 5·5; for 1886, 6·1; for 1877, 6·7; for 1888, 8·2; and for 1889, 9·6.

One result of this ready disparagement of vaccination has been to cause a slackness in the administration of the law with regard to it. Parents convicted of having neglected to have their children vaccinated are fined, and there the matter ends; no precaution is taken to see that the vaccination is performed after the fine has been paid, and those who can afford the guinea think they get off pretty well. The poor, whose neglect is more often due to ignorance than to disapproval, may be sent to prison, but very rarely the well-to-do. Yet vaccination is supposed to be compulsory and compliance with the Vaccination Act to be enforced by law. Doubtless there are many, on the Bench as well as elsewhere, who are now and then inclined to echo the opinion that "the law is a hass." If so, they are perfectly free to use their best endeavour to persuade the law to adopt less asinine ways, but when they have taken service under the law they are in duty bound to administer their master's orders, without evasion and without excuse. It would be curious to know how many of the unvaccinated remain so because of the leniency or indifference of magistrates.

It would be curious to know how many of those registered as "insusceptible" are so by nature, and how many by art. Some anti-vaccinators pride themselves on distributing tracts telling how the action of the virus may be nullified, and a child may escape as insusceptible. Ignorant mothers who object to the "sore arm" are tempted by these instructions, and doctors are cheated into giving what is really a false certificate. In some cases, no doubt, doctors themselves perform the operation hastily and carelessly, and by consequence unsuccessfully. It is noteworthy that they seemed to have no "insusceptibles" among the children who were vaccinated at the animal vaccine station at Lamb's Conduit Street, where calf lymph is cultivated for distribution among medical practitioners. Dr. Cory, in his report on the operations there, says: "Of the 7,529 primary vaccinations, 81 were of persons who failed to return for inspection; and of the remaining 7,448, all but 18 succeeded at the first attempt, and in no case was a third attempt at vaccination requisite." We may safely infer from this that with the employment of good lymph and a careful operator vaccination should never fail.

